# An Index of Surgery

**Published:** 1885-12

**Authors:** 


*An Index of Surgery.* By C. B. KEETLEY. Third Edition. Pp. 506. London: SMITH, ELDER, & Co. 1885.

We have already had occasion favourably to notice this work, and the early call for a third edition does not surprise us. The author is wise in eliminating, as well as in adding, and we congratulate him on his success in both directions.

In these busy times there is obtruded on us the uncomfortable question: Is the English language too prolix? Is stenography as feasible in composition as in penmanship? Are verbs and pronouns and adjectives necessities or luxuries in writing? When Mr. Keetley succeeds in conveying in a dozen words the meaning that others would spread over half a dozen sentences, we cannot help doubting the full value of the usual estimates put upon English grammar and rhetoric. It looks as if facts were too abundant and language too much drawn out for the modern student of medicine. In the interests of cerebration and of language we could wish that neither position was tenable: we are afraid that both positions are assured.

